# Breast cancer stem cells generate immune-suppressive T regulatory cells by secreting TGFβ to evade immune-elimination

**DOI:** 10.1007/s12672-023-00787-z

**Published:** 2023-12-01

**Authors:** Sumon Mukherjee, Sourio Chakraborty, Udit Basak, Subhadip Pati, Apratim Dutta, Saikat Dutta, Dia Roy, Shruti Banerjee, Arpan Ray, Gaurisankar Sa, Tanya Das

**Affiliations:** 1 Division of Molecular Medicine, Bose Institute, P-1/12, Calcutta Improvement Trust Scheme VII M, Kolkata, 700054 India; 2 Department of Pathology, ESI-PGIMSR, Medical College Hospital and ODC (EZ), Kolkata, India

**Keywords:** Anti-tumor immunity, Cancer stem cells, Metastasis, Microenvironment, Relapse, Stemness markers, TGFβ, Treg cells, Tumor initiation

## Abstract

Cancer stem cells (CSCs), being the primary contributors in tumor initiation, metastasis, and relapse, ought to have seminal roles in evasion of immune surveillance. Tumor-promoting CD4^+^CD25^+^FOXP3^+ ^T-regulatory cells (Tregs) have been described to abolish host defense mechanisms by impeding the activities of other immune cells including effector T cells. However, whether CSCs can convert effector T cells to immune-suppressive Treg subset, and if yes, the mechanism underlying CSC-induced Treg generation, are limitedly studied. In this regard, we observed a positive correlation between breast CSC and Treg signature markers in both in-silico and immunohistochemical analyses. Mirroring the conditions during tumor initiation, low number of CSCs could successfully generate CD4^+^CD25^+^FOXP3^+^ Treg cells from infiltrating CD4^+^ T lymphocytes in a contact-independent manner. Suppressing the proliferation potential as well as IFNγ production capacity of effector T cells, these Treg cells might be inhibiting antitumor immunity, thereby hindering immune-elimination of CSCs during tumor initiation. Furthermore, unlike non-stem cancer cells (NSCCs), CSCs escaped doxorubicin-induced apoptosis, thus constituting major surviving population after three rounds of chemotherapy. These drug-survived CSCs were also able to generate CD4^+^CD25^+^FOXP3^+^ Treg cells. Our search for the underlying mechanism further unveiled the role of CSC-shed immune-suppressive cytokine TGFβ, which was further increased by chemotherapy, in generating tumor Treg cells. In conclusion, during initiation as well as after chemotherapy, when NSCCs are not present in the tumor microenvironment, CSCs, albeit present in low numbers, generate immunosuppressive CD4^+^CD25^+^FOXP3^+^ Treg cells in a contact-independent manner by shedding high levels of immune-suppressive Treg-polarizing cytokine TGFβ, thus escaping immune-elimination and initiating the tumor or causing tumor relapse.

**Supplementary Information:**

The online version contains supplementary material available at 10.1007/s12672-023-00787-z.

## Introduction

Despite significant advances in cancer treatment, a major proportion of malignancies relapse thereby significantly increasing patient mortality [1]. To comprehend the cause of growing malignancies, understanding the tumor microenvironment (TME) that comprises not only tumor cells but also various immune cells, endothelial cells, fibroblasts, pericytes, and mesenchymal cells, as well as their concerted interaction leading to an effective tumor growth, becomes necessary [2]. During the past few decades, a myriad of evidences has proposed cancer stem cell (CSC) theory wherein, tumor initiation, growth, and progression are powered by a small sub-population of cells in the TME, termed CSCs [3]. CSCs furnish stem cell-like characteristics such as self-renewal, drug-resistance, and have been identified as the origin of carcinogenesis [4]. CSCs have also been linked to invasiveness and metastatic potential of any tumor [5]. Previous report from our laboratory identified CXCR4^+^ CSCs as the root cause of metastasis [6], which infiltrate to the surrounding region and initiate new tumors in secondary organs. CSCs also have the potential to migrate to distant locations and generate metastases that appear several years after therapeutic surgical treatment of a primary tumor [7, 8]. Moreover, despite an initial positive response to existing anti-cancer treatments, a significant proportion of patients relapse after a few months to several years [9, 10] since such therapeutic regimens can only eradicate highly proliferative non-stem cancer cells (NSCCs) that form the tumor mass, while highly resistant tumor-initiating CSCs survive [11]. As a result, although initially a decrease in tumor size is observed, CSCs, due to their stem-like qualities, might again form the heterogeneous cancer cell types thus triggering recurrence [12]. All the above metrics, point towards this tiny subset of tumor-initiating cells as the primary contributors to tumor initiation, metastasis, and even recurrence after therapy.

It is acknowledged that tumor niche contains a variety of immune cells that eliminate tumor during initial ‘elimination’ phase of tumorigenesis. To initiate tumor, either during primary initiation or during metastasis at secondary organ/tissue as well as during relapse, CSCs must, therefore, first overcome resistance from the host immune system and develop an immuno-suppressive microenvironment [13] to create, propagate and maintain the tumor. CSCs might therefore, modulate its anti-tumor immune-environment to pro-tumor one [14, 15], although the mechanism underlying the survival of CSCs during the ‘elimination phase’ of immune attack still remains elusive. It is well recognized that during tumor initiation, one of the key players of anti-tumor immunity i.e., naïve T cell, is primed in draining lymph nodes, get activated, and elicit an effector immune response at the tumor-initiation site [16]. As a fight-back mechanism, tumor cells produceCD4^+^CD25^+^FOXP3^+^ T-regulatory (Treg) cells from CD4^+^T cells [17, 18] by shedding multitude of chemokines [19]. These tumor Treg cells exhibit an indispensable role in inhibiting anti-tumor immunity [20] while promoting EMT, angiogenesis and invasion [21]. Elevated number of Treg cells have been found at the tumor site in patients [22]. It is also well accepted that in several cancers, plentiful Treg cells infiltrate into the tumor site resulting in poor clinical prognosis [23]. Evidences also suggest that tumor-derived TGFβ is one of the major drivers for induction of CD4^+^CD25^+^FOXP3^+^ Treg cells from CD4^+^CD25^−^ T cells [24]. However, there is hardly any report demonstrating the role of CSCs, if any, in Treg cell generation during ‘early’ tumor initiation phase when even NSCCs are not present in the microenvironment [25].

Here, our *in silico* and breast tumor patient tissue data showed a positive correlation between occurrence of CSCs and Treg cells. Our experiments mimicking the condition of tumor initiation when only a low number of breast CSCs are present in comparison to the infiltrating effector CD4^+^T cells, revealed that CSCs convert CD4^+^CD25^−^ T cells into immunosuppressive CD4^+^CD25^+^FOXP3^+^ Treg cells in a contact-independent manner. Moreover, unlike breast NSCCs, CSCs not only survive chemotherapeutic stress, but also, increase their own repertoire, thereby eventually culminating in enhanced Treg cell generation to ensure immune-evasion and subsequent tumor recurrence after chemo-withdrawal. Our search for the underlying mechanism further unveiled the role of CSC-shed immunosuppressive cytokine TGFβ, in generating tumor Treg cells. Therefore, our findings bolster our hypothesis that CSCs ensure their own survival by modulating tumor microenvironment in their favor, thus escaping immune-elimination, and initiating new tumor.

## Materials and methods

### Patient sample

Inclusion criteria of patient samples: 18 female patients of 18 to 65 years of age diagnosed with Breast Cancer (BC) with known ER/PR/HER2 and Neo-adjuvant chemotherapy (NACT) status were collected from ESI Post-Graduate Institute of Medical Science and Research in Kolkata, India. All the patients provided informed consent in accordance with the Helsinki Declaration. Specimens were collected from breast tumor patients undergoing surgery and processed within one hour of surgery. For collecting peripheral blood and tumor tissue samples from patients with breast tumor after surgery, ethical approval was taken from the ethics committees of the ESI Post-Graduate Institute of Medical Science and Research in Kolkata, India (Approval No: ESI-PGIMSR/MKT/IEC/13/2017, dated: 22 Dec 2017), and the Human Ethics Committee of the Bose Institute (Approval No: BIHEC/2017-18/7, dated: 28 May 2017). Online Resource 1 (Supplementary Table 1) contains all patient-related information.

### Cell culture

#### Adherent cell culture

Human breast carcinoma cell lines MCF-7, MDA-MB-231, and MDA-MB-468 were procured from National Centre for Cell Science, Pune, India and were routinely maintained in RPMI 1640 medium (Himedia®) with 10% (v/v) FBS (Thermofisher Scientific, Catalogue number 16000069) at 37 °C in a humidified incubator having 5% CO_2_ as previously described [6, 8]. After resuscitation, cells were maintained for less than six months. Cells were allowed to reach confluence before use.

#### Mammosphere culture

Adherent cells were trypsinized, washed with 1X PBS (Himedia^®^) and were plated at 2.5 × 10^4^ cells/well density (or as required) on six-well ultra-low adherence plates (Corning) in DMEM/F12 (Himedia^®^) with B27 supplement (BD Biosciences), recombinant epidermal growth factor (20 ng/ml) (Himedia^®^), basic fibroblast growth factor (20 ng/ml) (Himedia^®^), bovine insulin (5 µg/ml) (Sigma-Aldrich), and 0.4% bovine serum albumin (BSA) (Himedia^®^) as described earlier [6, 8]. Primary spheres were trypsinized and dissociated every 7 days followed by replating in serum-depleted media at 2.5 × 10^4^ cells/well onto ultra-low adherence six-well plates for secondary sphere formation. Phase-contrast images of secondary mammospheres were captured using Olympus IX83 microscope at 10X magnification.

### Purification of CSC population

CSCs (CD44^+^/CD24^−^) were isolated from tumor tissue or breast cancer cell lines using CD44 (MitenyiBiotec, CA, Cat. No. 130-095-194) and CD24 (MitenyiBiotec, CA, Cat. No. 130-095-951) MACS separation beads according to the manufacturer’s protocol. Isolated cells were maintained in serum-free DMEM/F12 media (Himedia^®^) supplemented with 4 µg/mL Insulin (Sigma), 20 ng/mL EGF (Himedia^®^), FGF (Himedia^®^) and 1:50 B-27™ supplement (BD Biosciences) 0.4% BSA (Himedia^®^) on low-adherence plates (Corning). NSCC population (CD44^−^/CD24^−^, CD44^−^/CD24^+^, CD44^+^/CD24^+^) from breast carcinoma cell lines was isolated using CD44 and CD24 MACS separation beads according to manufacturer’s instruction and further, cultured using complete DMEM media. Conditioned media (CM) from CSCs was collected after 72 h for further experiments.

### Treatment of cells

In experiments involving chemotherapy, MDA-MB-468 cells and corresponding CSCs were subjected to 2.5 µM doxorubicin (MP Biomedicals, CA) treatment for 24 h. After that, cells were replenished with fresh media. For consecutive 3-cycles of chemotherapeutic treatment, 2.5 µM doxorubicin was added to the CSC-culture media at an interval of 48 h [26].

### Immune subset

Peripheral blood from tumor patients was mixed with lymphocyte separating medium (HiSep, Himedia, Cat. No. LS001) in 1:1 ratio, and centrifuged for 40 min at 1000 rpm at room temperature. After collecting the buffy layers cells were washed and resuspended in sterile 1X PBS to obtain total lymphocytes. Naïve CD4^+^ T cells from total lymphocyte was purified with human CD4^+^ naïve T cell isolation kit (MojoSort, BioLegend, Cat. No. 480041) according to the manufacturer’s protocol. Purity of the isolated cells was determined flow-cytometrically. For Treg isolation as well as, monitoring Th1 response, naïve CD4^+^ T cells were cultured for 72 h in CSC-spent medium in the presence of anti-CD3/anti-CD28 (0.5 µg/ml each) (BD Pharmingen, Cat. No. 551916 and 556620, respectively) antibodies. Generated Treg cells were then purified based on negative CD127 expression and CD25 positive fraction using biotin mouse anti-human CD127 (BD Pharmingen, Cat. No. 558633) and anti-human CD25 Magnetic particles-DM (BD Imag, Cat. No. 558005), yielding consistently ~ 80% pure CD4^+^CD25^+^CD127^−^ Treg cells as determined by flow-cytometry (Online Resource 3, Supplementary Fig. 1E). Purified cells were grown in RPMI-1640 supplemented with L-glutamine and NaHCO_3_ (Sigma-Aldrich, Cat. No. R8758) and 10% (v/v) FBS (fetal bovine serum) (Thermofisher Scientific, Cat. No. 16000069), as well as penicillin-streptomycin in a humidified CO_2_ incubator at 37 ^o^C. (Thermofisher Scientific, Cat. No. 15140122). To generate classical T regulatory cells as control, isolated naïve T cells were activated with anti-CD3/anti-CD28 and incubated with TGFβ (5 ng/ml) and IL2 (50 U/ml) [27] (Peprotech, Cat. No. 100-21, and 200-02 respectively) for 72 h. For monitoring Th1 response under TGFβ influence, activated CD4^+^ T cells were treated with recombinant TGFβ (5ng/ml) for 72 h.

### Flow cytometry

The fluorochrome-tagged antibodies against human proteins used in flow-cytometry are: CD4-APC-H7/-FITC, CD25-PE-Cy7, FOXP3-APC, CD127-APC/-PerCP-Cy5.5, TGFβ1-BV421, CD44-APC, CD24-PE, Annexin V-FITC, OCT4-PerCP-Cy5.5, SOX2-APC, NANOG-PE. All antibody details are listed in Online Resource 2 (Supplementary Table 2).

For multi-color flow-cytometric surface labelling, cells were tagged with specific antibodies and incubated at 4 °C for 30 min. For staining of intracellular FOXP3 protein, transcription factor buffer (BD Pharmingen, Cat. No. 562574) was used to fix and permeabilize the cells. Cells were incubated with the cell activation cocktail (Ionomycin and PMA) in presence of brefeldin-A (Biolegend, Cat. No. 423304) for the last 5 h at 37 °C to identify the intracellular cytokines TGFβ and IFNγ. The cells were then washed, fixed, and permeabilized with the cytofix and cytoperm buffer combination (BD Biosciences, Cat. No. 554714), after which staining was done for 30 min with anti-IFNγ-PE and anti-TGFβ-BV421. For intra-cellular staining of OCT4, SOX2, and NANOG; BDStemflow Pluripotent Stem Cell Transcription Factor Analysis Kit (Cat. No. 560589) was used as per manufacturer’s protocol. Cell apoptosis was assessed flow-cytometrically, using FITC AnnexinV apoptosis detection kit (BD Pharmingen, Cat. No. 556547) as per the manufacturer’s instruction. After specific treatment, cells were harvested and washed with PBS, resuspended in Annexin-V binding buffer, and incubated with anti-Annexin-V-FITC antibody for 45 min [28]. The data was collected using BD FACSVerse, and it was analyzed using BD FACSVerse Suite software (BD Biosciences). To establish the quadrant gates and to measure stained cells in contour plots, dot plots, appropriate isotype controls as well as fluorescence minus one (FMO) were used.

### TGFβ neutralization assay

For this assay, MDA-MB-468 cell-derived CSC-CM was preincubated with anti-TGFβ antibody (BD Pharmingen, Cat. No. 555052) at a concentration of 1 µg/ml for neutralizing TGFβ. After that, CD4^+^ T lymphocytes were cultured with anti-CD3/anti-CD28 antibodies for further 72 h with or without the afore-mentioned CM.

### ELISA assay

To evaluate the production of TGFβ, the Human TGF-beta 1 ELISA kit (Ray Biotech, Cat. No.ELH-TGFb1) was used. For that, spent media from MDA-MB-468-derived NSCCs and CSCs vis-à-vis doxorubicin-treated or untreated MDA-MB-468 cell-derived CSCs were harvested and analyzed for TGFβ. ELISA were performed as per the manufacturer’s specifications (RayBiotech) and the absorbance was read at 450 nm using ThermoScientific MultiskanGO instrument. A standard curve of TGFβ was plotted using kit-supplied TGFβ standards.

### The ex-vivo T-cell suppression assay

From CSC-CM-induced T cells (isolated from breast tumor patients’ blood), CD4^+^CD25^+^CD127^−^ Treg cells (0.5 × 10^5^ cells) were sorted by Magnetic bead sorter, and were co-cultured with purified autologous naïve T cells (1 × 10^5^ cells) in presence of anti-CD3/anti-CD28 antibodies for 72 h. IFNγ positivity in activated T cells was determined flow-cytometrically from each experimental set. Similarly, proliferation of CD4^+^ T-cells was evaluated by CFSE (Biolegend, Cat. No. 423801) dilution assay. For CFSE assay, cells were stained with 5µM CFSE in PBS for 20 min at 37 °C. The cells were then resuspended in 10% (v/v) FBS containing complete media and washed twice with 1X sterile PBS. The CFSE-labelled T cells were either resuspended in complete media directly or plated with CSC-induced Treg at a 1:2 ratio for 72 h at 37 °C and 5% CO_2_. The percent CFSE^+^ T cells was calculated by a FACSVerse cytometer [29].

### RNA isolation and quantitative real-time PCR amplification

Total cellular RNA extraction was done with RNAiso Plus reagent (Cat. No. 9108, Takara) and 1µgof that RNA was reverse transcribed using PrimeScript 1st strand cDNA synthesis kit (Cat. No. RR037A, Takara). Quantitative Real Time PCR (qRT-PCR) was performed with TB Green Premix Ex Taq SYBR Green master mix (Cat. No. RR820A, Takara) in Roche Light Cycler 96 system, using that cDNA [30]. The target gene amplification was performed with the following primers specific for OCT4 (forward: 5′-GGGCTCTCCCATGCATTCAAAC-3′; reverse: 5-′CACCTTCCCTCCAACCAGTTGC-3′), SOX2 (forward: 5′-CCATGCAGGTTGACACCGTTG-3′; reverse: 5′-TCGGCAG-ACTGATTCAAATAATACAG-3′), NANOG (forward: 5′-AGTCCCAAAGGCAAACAACCCACTTC-3′; reverse: 5′-TGCTGGAGGCTGAGGTATTTCTGTCTC-3′), ALDH1A1 (forward: 5′-CGTGGCGTACTATGGATGCT-3′; reverse: 5′-TGGAT-CTTGTCAGCCCAACC-3′), N-CADHERIN (forward: 5′-AGTCCCAAAGGCAAACAACCCACTTC-3′; reverse: 5′-TGCTGGAGGCTGAGGTATTTCTGTCTC-3′), VIMENTIN (forward: 5′-TCTCTGAGGCTGCCAACCG-3′; reverse: 5′-CGAAGG-TGACGAGCCATTTCC-3′), ABCG2 (forward: 5′-CTTACAGTTCTCAGCAGCTCTTCG-3′; reverse: 5′-CGAGGCTGATGAATGGAGAAG-3′), MRP1 (forward: 5′-ACCATCCACGACCCTAAT-3′; reverse: 5′CCACCTTGGAACTCTCTTTC-3′), MDR1 (forward: 5′-GGAGATAGGCTGGTTTGATG-3′; reverse: 5′-GTCCAAGAACAGGACTGATG-3′), β-ACTIN (forward: 5′-AGGTCATCACCATTGGCAAT-3′; reverse: 5′-ACTCGTCATACT-CCTGCTTG-3′),

GAPDH (forward: 5′-CCTGCACCACCAACTGCTTA-3′reverse: 5′-GGCCATCCACAGTCTTCTGGG3), 18S rRNA (forward: 5′-GGAATTGACGGAAGGGCAC-3′; reverse: 5-′CGCTCCACCAACTAAGAACG-3′). For quantitative real-time PCR, relative mRNA expression was determined using ∆∆Ct technique with 2^−∆∆Ct^ as fold change [31].

### Immunohistochemistry

Tissues were taken from the resected breast tumor immediately after surgery. Specimens were formalin-fixed, processed, and embedded on paraffin. 4 μm thick tissue-sections were cut on a microtome (Leica). Formalin-fixed tissues were deparaffinized and rehydrated gradually before antigen retrieval in TRIS-EDTA buffer (PH-9.0). Samples were then subjected to peroxidase treatment, blocked in 2% (w/v) BSA followed by overnight incubation with primary antibody at 4 °C. Primary antibody (1:100) against OCT4, SOX2, NANOG, and FOXP3 (Thermofisher Scientific, Cat. No. MA1104, MA1014, MA1017, and PA1806, respectively) were used. After washing, slides were incubated with diluted (1:500) secondary antibody (Santacruz Biotech, Cat. No. 516102 and Cat. No. 2357, respectively) for 1 h, treated with the chromogenic substance 3,3′-diaminobenzidine (Abcam, Cat. No. ab64238), and thereafter counter-staining was done in hematoxylin (SRL, India). The slides were washed, dehydrated, and finally mounted in DPX (Himedia, India). Images were acquired in a Leica bright-field microscope [32].

### Survival, correlation, and expression analyses

Overall survival (OS) probability and relapse free survival (RFS) probability of breast tumor patients involving OCT4, NANOG, and FOXP3 were executed using Kaplan-Meier plotter (https://kmplot.com) [33–35]. ‘R2: Genomics Analysis and Visualization Platform’ (http://r2.amc.nl) was used to map correlation using publicly available datasets between NANOG:FOXP3, OCT4:FOXP3 in breast cancer (dataset used:GSE25066), ALDH1A1:FOXP3 in brain cancer glioma (dataset used: Tumor Brain Lower Grade Glioma (2022-v2)-tcga-532-tpm-gencode36) and ALDH1A1:FOXP3 in prostate cancer (dataset used: Tumor Prostate Adenocarcinoma - TCGA − 497 - rsem–tcgars) (r = 0.1529, p < 0.001, n = 497). The same website was used to make correlation plots between ALDH1A1: TGFβ1 and TGFβ1: FOXP3 in breast cancer using dataset GSE69031 and GSE5460, respectively. The mRNA expression of OCT4, SOX2, NANOG, and ALDH1A1 as well as FOXP3 was analyzed using the publicly available RNA profiling dataset (GSE5460) for ER^+^ luminal breast cancer and dataset (GSE30682) for triple negative breast cancer (TNBC) using ‘R2: Genomics Analysis and Visualization Platform’. TNBC dataset Tumor TNBC Breast (Brain metastasis)-Biernat-71-custom-ilmnht12v4r2w (GSE76714) from ‘R2: Genomics Analysis and Visualization Platform’ was used to map correlation between CSC-related stemness factor ALDH1A1 and the Treg signature gene FOXP3 in TNBC. Similarly, another TNBC dataset Tumor Breast (triple negative)-Purrington-226-rma_sketch-hugene21t (GSE142102) from ‘R2: Genomics Analysis and Visualization Platform’ was utilized to assess correlation between ALDH1A1 and TGFβ, along with TGFß and FOXP3 in TNBC.

### Statistical analysis

For all statistical analyses, GraphPad Prism 9.3.1. was used unless otherwise noted. All data are the mean from a minimum of three independent experiments if not mentioned otherwise. Data with normal distribution were analyzed by either Student’s *t* test or analysis of variance (ANOVA) as appropriate. For multiple comparisons where applicable, one-way ANOVA was performed for normal data and resulting significant (p *< 0.05)* p values have been furnished.

## Results

### Tumor-initiating CSCs are positively correlated with immunosuppressive Treg cells in breast tumor tissue

**Fig. 1:**
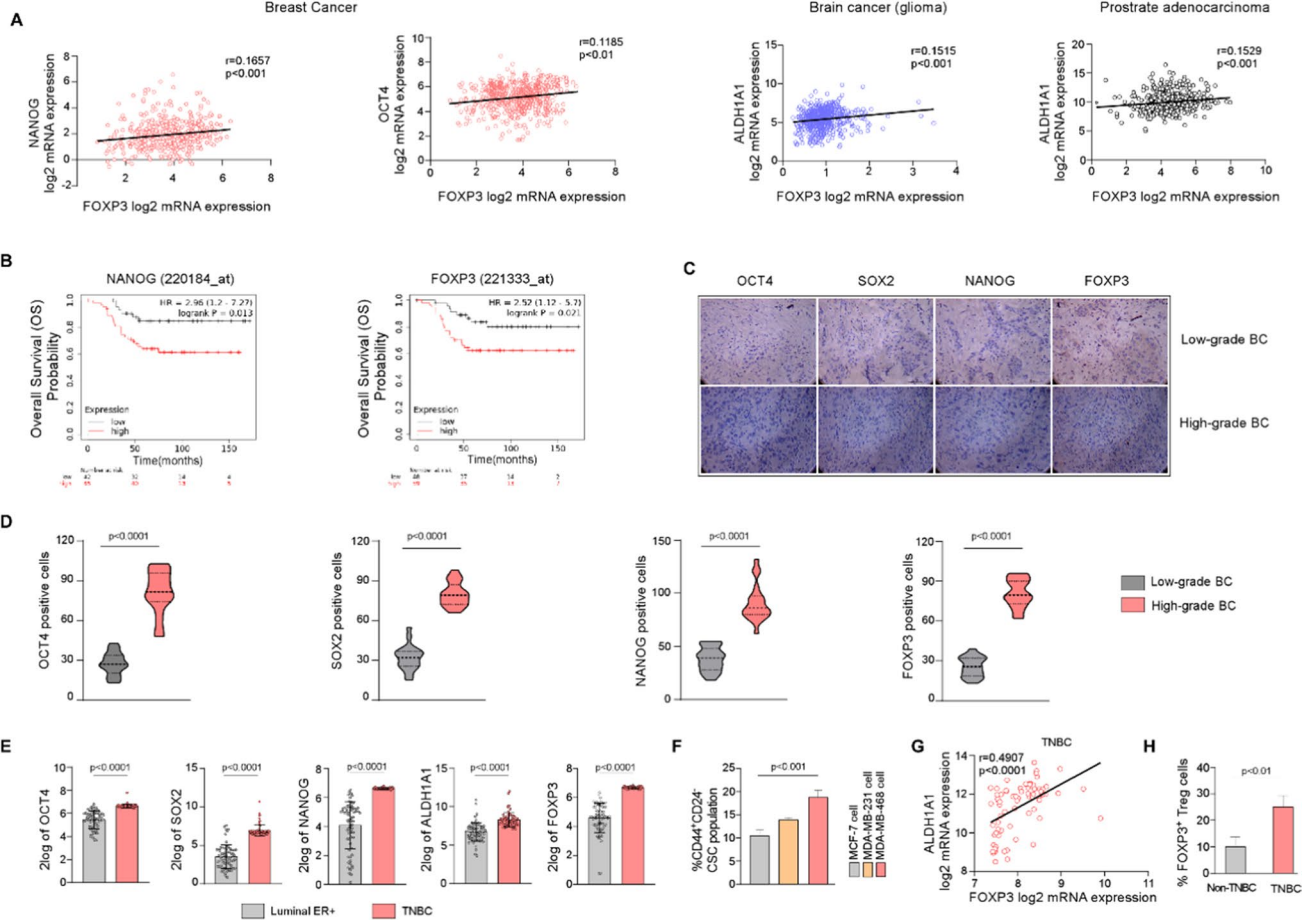
Tumor-initiating CSCs and immunosuppressive Treg cells have positive correlation in breast cancer. **A** Plots showing correlation between NANOG and FOXP3, OCT4 and FOXP3, from GSE25066 breast cancer patient dataset (left). Plots demonstrating positive correlation between cancer stemness marker ALDH1A1 and suppressive Treg signature FOXP3 in brain cancer glioma (dataset used: Tumor Brain Lower Grade Glioma (2022-v2)-tcga-532-tpm-gencode36) (middle) and prostate cancer (dataset used: Tumor Prostate Adenocarcinoma-TCGA-497-rsem-tcgars) (right). Datasets were obtained from ‘R2: Genomics Analysis and Visualization Platform’ and ‘TCGA’ database. “r” is correlation co-efficient. **B** Kaplan-Meier (KM) plot depicting low overall survival (OS) probability of breast tumor patients harboring high expression of CSC marker NANOG and Treg signature gene FOXP3. **C** Representative Immunohistochemistry (IHC) images showing elevated expression of stemness markers OCT4, SOX2, NANOG, and Treg signature gene FOXP3 in low-grade vs. high-grade breast tumor tissues (n = 4 in each group). Scale bar = 50 µM and magnification 40X. **D** Bar diagram showing number of OCT4, SOX2, NANOG, and FOXP3 positive cells in low-grade vs. high-grade breast tumor tissues as determined by IHC-staining. **E** Bar graphs showing incidence of higher cancer stemness markers OCT4, SOX2, NANOG, and ALDH1A1 along with suppressive Treg marker FOXP3 in TNBC tissues than ER^+^-luminal breast tumor tissues. Bar graphs were plotted using RNA profiling data of TNBC dataset (GSE30682) and ER^+^-luminal breast tumor dataset (GSE5460) available in ‘R2: Genomics Analysis and Visualization Platform’. **F** Flow-cytometry data showing percent CD44^+^CD24^−^ CSC population in ER^+^-luminal MCF-7, triple-negative MDA-MB-231, and MDA-MB-468 breast cancer cells. **G** Plot showing a positive correlation between ALDH1A1 and FOXP3 in TNBC dataset (GSE76714) available in ‘R2: Genomics Analysis and Visualization Platform’. **H** Bar graph demonstrating occurrence of higher Treg cell percentage in TNBC patients than non-TNBC patients. Data were represented as the mean ± SD of minimum 3 independent experiments performed in triplicate. Student’s *t*-test (unpaired) (**D**, **E**, **H**) and one-way ANOVA (**F**) was used to assess the data where *P < 0.05, **P < 0.01, ***P < 0.001, and ****P < 0.0001. BC: breast cancer, TNBC: triple-negative breast cancer

To evaluate the relationship between breast CSCs and Tregs, if any, we undertook a multipronged approach to obtain cross-validating datasets. To that end, in our first approach, our in-silico study based on RNA-profiling dataset (GSE25066) available from ‘R2: Genomics Analysis and Visualization Platform’ using CSC (OCT4 and NANOG) as well as, Treg (FOXP3) signatures revealed a positive correlation (r = 0.1185, p < 0.01; OCT4 and FOXP3) (r = 0.1657, p < 0.001; NANOG and FOXP3) (n = 508) between breast CSCs and Tregs (Fig. 1A left panel). We extended our observation to include information regarding other tumor tissues as well by mining publicly available tumor datasets from ‘R2: Genomics Analysis and Visualization Platform’ and ‘The Cancer Genome Atlas’ (TCGA). Data obtained were in line with our findings in breast tumor tissue, i.e., CSC (ALDH1A1) and Tregs (FOXP3) markers possessed a positive correlation in glioma (dataset: Tumor Brain Lower Grade Glioma (2022-v2) - tcga − 532 - tpm - gencode36) (r = 0.1515, p < 0.001, n = 532) and prostate adenocarcinoma (dataset: Tumor Prostate Adenocarcinoma-TCGA-497- rsem–tcgars) (r = 0.1529, p < 0.001, n = 497) (Fig. 1A right panel).

In our second approach, Kaplan-Meier survival curve revealed lower probability of overall survival (OS) in breast tumor patients with higher CSC (NANOG) (p < 0.05) and Treg (FOXP3) (p < 0.05) signature genes (Fig. 1B), in concordance with earlier reports [36–38]. Third, to broaden the scope of our data, we performed immunohistochemistry (IHC) of breast tumor patient-derived tissues, which revealed an augmented incidence of stemness factors (OCT4, SOX2, NANOG) along with Treg signature gene FOXP3 (Fig. 1C) in high-grade (n = 4) than low-grade (n = 4) breast tumor tissues. Moreover, we observed and calculated the number of OCT4/SOX2/NANOG/FOXP3 positive cells per area taken from sections of the tumor tissues (Fig. 1C), and consequently found similar trend in stemness genes and FOXP3 coincides with high-grade of breast tumor compared to low-grade breast tumor (p < 0.0001) (Fig. 1D). Thus, raising the possibility of a direct relationship between them due to their increased levels in breast cancer.

In our fourth approach, we employed RNA expression datasets from ‘R2: Genomics Analysis and Visualization Platform’ database to identify the breast cancer subtype possessing highest percent of CSCs. Our analysis revealed significantly higher CSC-related stemness genes (OCT4, SOX2, NANOG) (p < 0.0001) and ALDH1A1 (p < 0.0001) in TNBC (GSE30682), in contrast to ER^+^ luminal breast cancer (GSE5460) (Fig. 1E). Reflecting these results, when we checked the CSC contents of different breast cancer cell lines, we obtained higher percent of (CD44^+^/CD24^−^) CSCs in TNBC cell lines, i.e., MDA-MB-468 and MDA-MB-231, as compared to luminal MCF-7 cell line (Fig. 1F). Among these two TNBC cell lines, MDA-MB-468 furnished the highest percentage of CSCs (~ 20%) (Fig. 1F). Concurrently, ‘R2: Genomics Analysis and Visualization Platform’ database analysis demonstrated significantly higher FOXP3 expression (p < 0.0001) in TNBC (GSE30682), when compared with ER^+^ luminal subtype (GSE5460) (Fig. 1E, right panel) validating our results. To understand whether the mutuality between CSCs and Treg is reflected in TNBCs as well, we performed RNA profiling dataset analysis from ‘R2: Genomics Analysis and Visualization Platform’ database and observed a positive correlation between ALDH1A1 and FOXP3 in TNBC patient dataset (GSE76714) (p < 0.0001, r = 0.4907, n = 71) (Fig. 1G).

Since, the expressions of the gene signatures do not necessarily correlate with the actual number of cells, in our fifth approach, we directly evaluated the percentage of CD4^+^CD25^+^FOXP3^+ ^Tregs from breast tumor patient-derived blood samples. In line with our above findings, this experiment also revealed significantly higher Treg (p < 0.01) percentages in high-CSC-containing (Fig. 1E) TNBC patients’ (n = 4) blood as compared to non-TNBC patient (n = 4) cohort (Fig. 1H).

Such step-wise multi-approach investigations involving in silico and patient tissue analysis strongly indicated a direct relation between CSCs with Tregs.

### CSCs, even in low numbers, are able to generate Treg cells

**Fig. 2:**
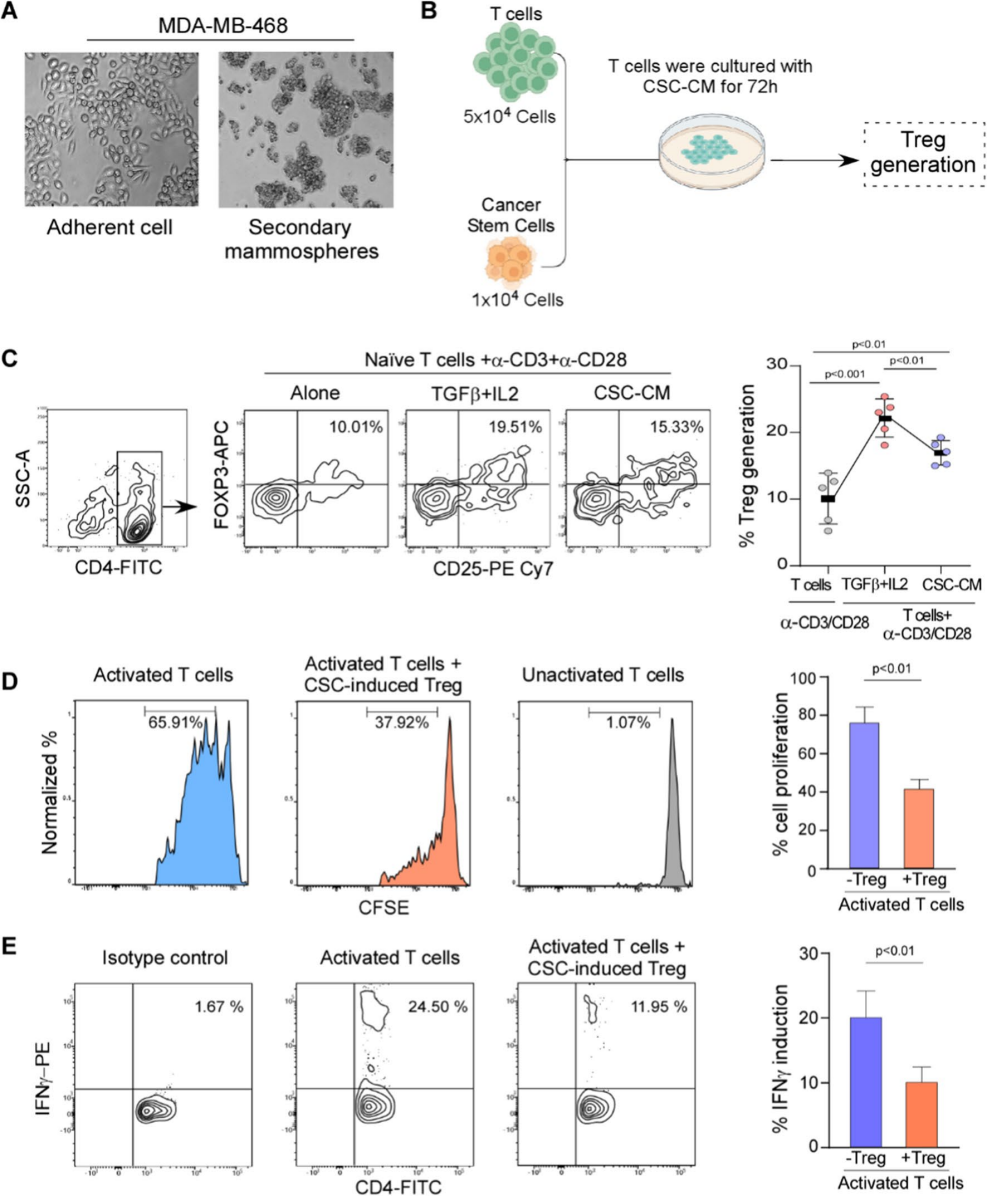
Low number of CSCs are sufficient to generate immunosuppressive Treg cells. **A** Inverted microscopic image showing the appearance of monolayer MDA-MB-468 cells and cell-derived spheroids viewed under 10X magnification. **B** Schematic representation of immunosuppressive Treg cell generation using CSC-CM taking single-cultured CSCs and T cells in 1:5 ratio. **C** FACS plots (left panel) and representative graph (right panel) showing immunosuppressive Treg cell percentage in (i) only α-CD3 + α-CD28, (ii) α-CD3 + α-CD28 + TGFβ + IL2, and (iii) α-CD3 + α-CD28 + CSC-CM. **D** Flow-cytometric histoplots (left panel) and representative bar graph (right panel) showing T-cell proliferation in presence of CSC-induced Treg cells. **E** FACS plots (left panel) and bar graph (right panel) furnishing percentage of IFNγ secreting CD4^+^ T cells after co-culture with CSC-induced Treg cells. Data were represented as the mean ± SD of minimum 3 independent experiments performed in triplicate. Student’s t-test (unpaired) was used to assess the data where *P < 0.05, **P < 0.01, ***P < 0.001, and ****P < 0.0001. CSCs: cancer stem cells; CM: conditioned medium

Above results indicating a direct relationship between CSCs and Tregs in breast tumor tempted us to explore as to how during tumor initiation, CSCs, although present in low number [39] in comparison to surrounding anti-tumor effector CD4^+^ T cells, not only evade immune-elimination but also proliferate and form the entire tumor mass by generating NSCCs. To that end, human TNBC cell line MDA-MB-468 was utilized for generating CSC-enriched secondary mammospheres (Fig. 2A) since these breast cancer cells furnished highest CSC percentage (Fig. 1F). MDA-MB-468-derived secondary mammospheres generated ~ 3.5-fold CD44^+^/CD24^−^ CSC enrichment as compared to MDA-MB-468 cells (p < 0.0001) (Online Resource 3, Supplementary Fig. 1A). From these secondary mammospheres, breast CSCs were purified using Miltenyi bead isolation protocol (Schematic diagram depicted in Online Resource 3, Supplementary Fig. 1B). Flow cytometry data further confirmed > 90% purity of CD44^+^/CD24^− ^CSCs (Online Resource 3, Supplementary Fig. 1C). These CSCs were further characterized to obtain a significant higher expression of (i) stemness markers OCT4 (p < 0.001), SOX2 (p < 0.01), and NANOG (p < 0.01); (ii) EMT markers N-CADHERIN (p < 0.01), and VIMENTIN (p < 0.01), as well as (iii) drug resistance markers ABCG2 (p < 0.01), MRP1(p < 0.01), and MDR1 (p < 0.01) (Online Resource 3, Supplementary Fig. 1D), when compared with corresponding NSCCs (CD44^−^/CD24^−^, CD44^−^/CD24^+^, CD44^+^/CD24^+^). These results together establish that CD44^+/^CD24^−^ breast cancer cell subpopulation was indeed CSC in nature.

Considering the scenario that during initiation only a low number of CSCs remain in the site of tumor origin in comparison to higher number of infiltrating T cells [40, 41], next in an attempt to mimic such condition, anti-CD3/anti-CD28-activated CD4^+^ T cells (for 72 h) were cultured in the CM of 72 h culture of purified MDA-MB-468 CSCs, at the CSC:T cell ratio of 1:5, as depicted by the schematic diagram (Fig. 2B). Our flow-cytometry results (Fig. 2C, left panel) show that CM from even such low number of CSCs was able to generate significantly higher CD4^+^CD25^+^FOXP3^+^ Treg cells (p < 0.01) as compared to activated T cell counterpart (treated with anti-CD3/anti-CD28) (Fig. 2C, right panel). Activated T cells treated with IL2 and TGFβ1 to generate Treg were taken as positive control (Fig. 2C) [27, 42].

To evaluate the immunosuppressive properties of these newly generated Treg cells, we magnetically sorted CD4^+^CD25^+^CD127^−^ Treg cells (Online Resource 3, Supplementary Fig. 1E) since CD4^+^CD25^+^CD127^−^ Treg cells mostly correspond to CD4^+^CD25^+^FOXP3^+^ Treg cell population [43–45]. To that end, CD4^+ ^T cells were labelled with CFSE and co-cultured those with CSC CM-induced Treg cells. Results of Fig. 2D demonstrated significant inhibition (p < 0.01) in proliferation of the activated CD4^+ ^T lymphocytes in presence of the CSC-induced Treg cells. Furthermore, upon co-culturing with Treg fraction, CD4^+^ effector T cells showed significant decrease (p < 0.01) in IFNγ expression (Fig. 2E), which is a signature secretome of effector T cells [46].

All these findings together confirmed that tumor-initiating CSCs, despite of being present in low numbers as compared to the effector T cells, transformed the latter into immunosuppressive Treg cells in contact-independent manner to ensure escape from immune-elimination.

### Chemotherapy-spared CSCs induce Treg generation mimicking the condition of tumor initiation during relapse

Plethora of reports demonstrate the failure of conventional chemotherapy to kill CSCs [8, 47, 48]. Reports from our lab as well as others have further established the enrichment of CSC repertoire during chemotherapy [8, 49]. Furthermore, an enrichment of RNA transcripts of CSC-associated markers upon chemo-treatment was also noticed in TNBC that ultimately resulted in poor relapse-free survival (RFS) [50, 51]. In this relation, our Kaplan-Meier analyses revealed that NACT-treated breast cancer patients with elevated OCT4 and NANOG have significantly lower RFS (OCT4, p < 0.05; NANOG, p < 0.01) (Online Resource 4, Supplementary Fig. 2A and B) in comparison to patients furnishing lower levels of OCT4 and NANOG. These results as well as all above-mentioned reports tempted us to explore whether these chemo-escaped CSCs further generate immune-suppressed microenvironment thus subsequently causing tumor relapse after withdrawal of the treatment. For the same, we again undertook multi-approach experimentations as described below.

**Fig. 3:**
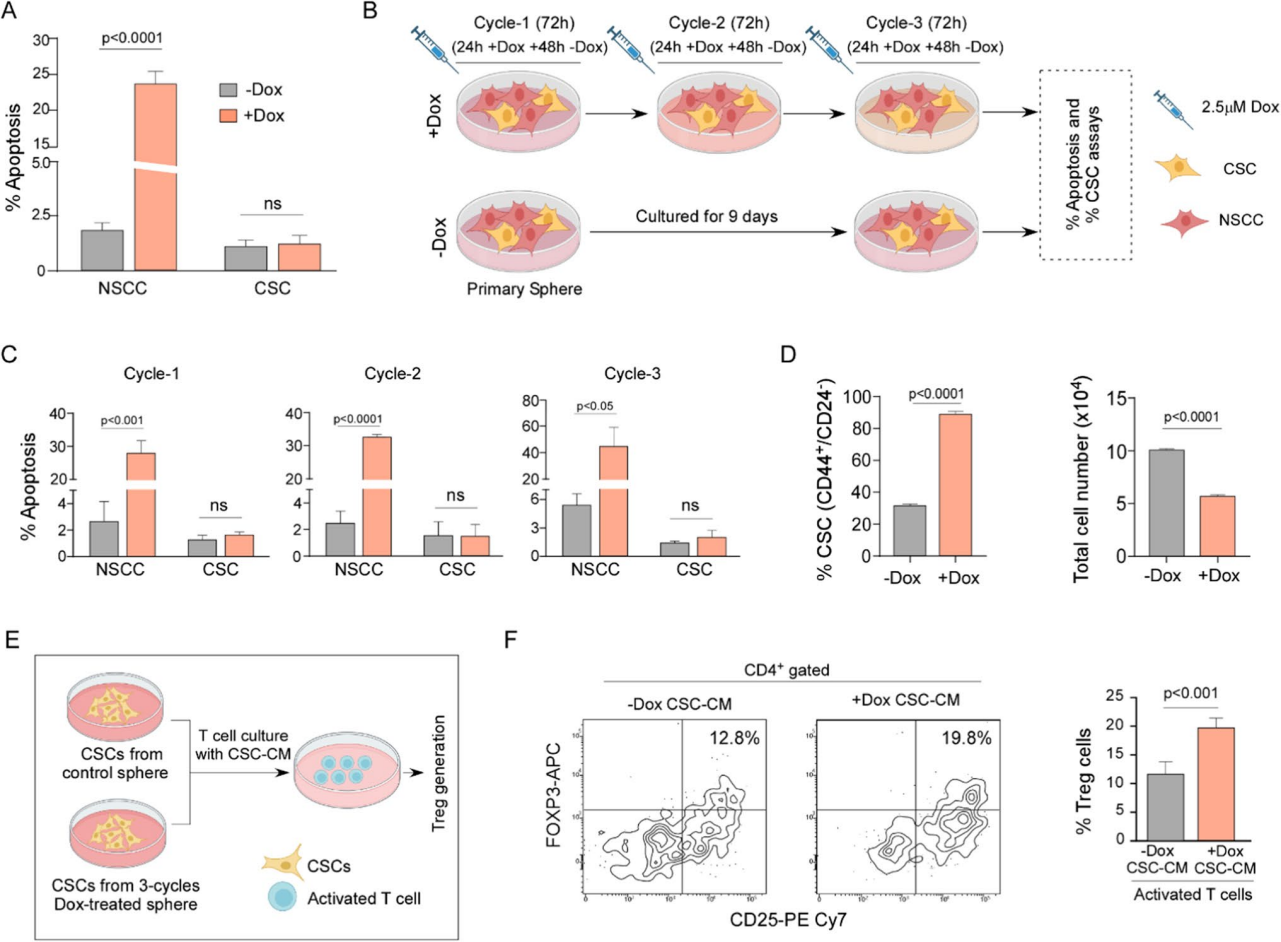
Doxorubicin-spared CSCs induce immunosuppressive Treg cells mimicking tumor-initiation following tumor relapse. **A** Bar graph showing percentage of apoptotic NSCCs and CSCs populations gated from MDA-MB-468 cells after doxorubicin treatment, as determined using flow-cytometry. **B** Schematic representation of three cycles of doxorubicin treatment regimen to MDA-MB-468 cell-derived spheroids. **C** Bar diagrams depicting percentage of apoptotic NSCCs and CSCs after 1st cycle (left), 2nd cycle (middle), and 3rd cycle (right) of chemotherapy. Percent apoptosis was flow-cytometrically determined using Annexin-V binding assay. **D** Representative bar diagram showing percentage of CD44^+^/CD24^−^ CSCs in control vs. post- 3 cycle doxorubicin-treated MDA-MB-468-derived spheroids (left). Bar plot showing total number of cells counted using hemocytometer from control vs. post-3 cycledoxorubicin-treated spheroids. **E** Schematic representation of Treg cell generation using MDA-MB-468 spheroid-derived CSC-CM and CSC-CM after 3 cycles of doxorubicin treatment. **F** Flow-cytometric plots showing percentage of Treg cells (left) and bar graph (right panel) showing percentage of suppressive Treg cells generated from activated T cells in presence of doxorubicin-treated or untreated MDA-MB-468 spheroid-derived CSC-CM. Data were represented as the mean ± SD of minimum 3 independent experiments performed in triplicate. Student’s t-test (unpaired) was used to assess the data where *P < 0.05, **P < 0.01, ***P < 0.001, and ****P < 0.0001. CSCs: cancer stem cells; Sph: spheroids; CM: conditioned medium

First, MDA-MB-468 cells were treated with doxorubicin (2.5 µM) for 24 h, that induced significant apoptosis in NSCC fraction (p < 0.0001) but failed to do so in CSCs (Fig. 3A). At this juncture, we considered the following information: (i) after multiple rounds of chemo-treatment, NSCCs are targeted and killed causing a decrease in tumor size while rare CSCs survive these therapeutic regimens, and ‘recreate’ the tumor after anti-cancer therapy [52, 53], either in the same or secondary organ; and (ii) increase in stemness markers depicting poor RFS (Online Resource 4, Supplementary Fig. 2A and B). These led us to hypothesize that such drug-surviving CSCs might be causing relapse after chemotherapy. Consequently, in our second approach, we treated MDA-MB-468 primary spheres (containing ~ 30% CSCs) with doxorubicin (2.5 µM) for 24 h then the treatment was removed and spheres were incubated in fresh media for another 48 h; this sequence was carried out for 3 cycles to mimic multiple chemotherapy sessions that may lead to relapse later (Schematic diagram Fig. 3B). Our results showed that NSCCs underwent significant apoptosis with each cycle (cycle 1 p < 0.001, cycle 2 p < 0.0001, cycle 3 p < 0.05) (Fig. 3C), whereas, CSCs could escape the treatment even after the last cycle of chemotherapy (cycle 3) since no apoptosis was noticed in this CSC fraction after each cycle (Fig. 3C). Our representative graph depicting cell number pre- and post- 3 cycles-treatment (Fig. 3D right panel), showed that while ~ 100,000 cells (30% CSCs and 70% NSCCs as seen in Fig. 3D left panel) were present before treatment, after the third cycle of treatment ~ 57,000 cells were alive. Among these chemo-escaped cells, 90% was CSCs and 10% NSCC (Fig. 3D left panel), indicating that the reduction in total cell number was due to killing of NSCCs, and majority of drug-surviving cells were CSCs. We next investigated the status of Treg cell generation after chemotherapy. To that end, in our third approach, in-silico Kaplan-Meier data of NACT-treated breast cancer patients showed positive correlation between higher FOXP3 expression (p < 0.05) (Online Resource 4, Supplementary Fig. 2C) with lower RFS, as compared to NACT patients expressing lower FOXP3, reflecting the correlation pattern we found with higher stemness (OCT4, NANOG) and lower RFS (Online Resource 4, Supplementary Fig. 2A and B) under similar conditions. Fourth, immunohistochemical analysis of breast cancer patient tissue samples revealed higher expression of CSC-related stemness genes (OCT4, SOX2, and NANOG) along with Treg marker FOXP3, in chemotherapy-treated cohort (n = 5), as compared to non-chemotherapy treated group (n = 5) (Online Resource 4, Supplementary Fig. 2D).

To validate the same, in our fifth approach, we isolated CSCs from untreated primary spheres and from the surviving population after 3rd cycle of chemotherapy. Interestingly, we observed fold increase in mean fluorescence intensity (MFI) of stemness factors Oct4 (p < 0.01), Sox2 (p < 0.0001), and Nanog (p < 0.0001) after the 3rd cycle chemotherapy-escaped CSC in contrast to untreated primary sphere-derived CSCs (Online Resource 4, Supplementary Fig. 2E), indicating an increase in stemness per CSC after 3 consecutive rounds of doxorubicin treatment. Next, anti-CD3/anti-CD28-activated CD4^+^ T cells were cultured in the CM collected from same numbers of CSCs from both the sets in a CSC: T cell ratio of 1:5, for another 72 h (Schematic diagram shown in Fig. 3E). Our results depicted significant immunosuppressive Treg generation (p < 0.001) from CM of CSCs isolated post-3 cycles of chemotherapy as compared to CSCs isolated from untreated MDA-MB-468-derived primary mammospheres (Fig. 3F).

Above-mentioned multi-pronged study establishes generation of immunosuppressive Treg cells by chemo-escaped CSCs during condition mimicking tumor relapse. Since chemotherapy itself causes immunosuppression [54], remaining T cells might be getting converted to Treg cells by drug-spared CSCs thereby aggravating that situation and ensuring survival of the drug-surviving CSCs for initiating tumor again thereby, causing tumor relapse after chemotherapy.

### CSCs generate Treg cells by secreting TGFβ

Next, we aimed at deciphering the mechanism underlying CSC-induced Treg cell generation. To that end our literature search revealed the crucial role of the cytokine TGFβ in Treg cell generation [55–57]. Further reports also specified the release of TGFβ by cancer cells [58, 59]. In line with this, murine pancreatic cancer cells reportedly converted naïve T cells to CD4^+^FOXP3^+^ Treg cells by releasing TGFβ [60]. This information together led us to hypothesize whether CSC-shed TGFβ is the molecule behind such generation of Treg cells during tumorigenesis.

**Fig. 4:**
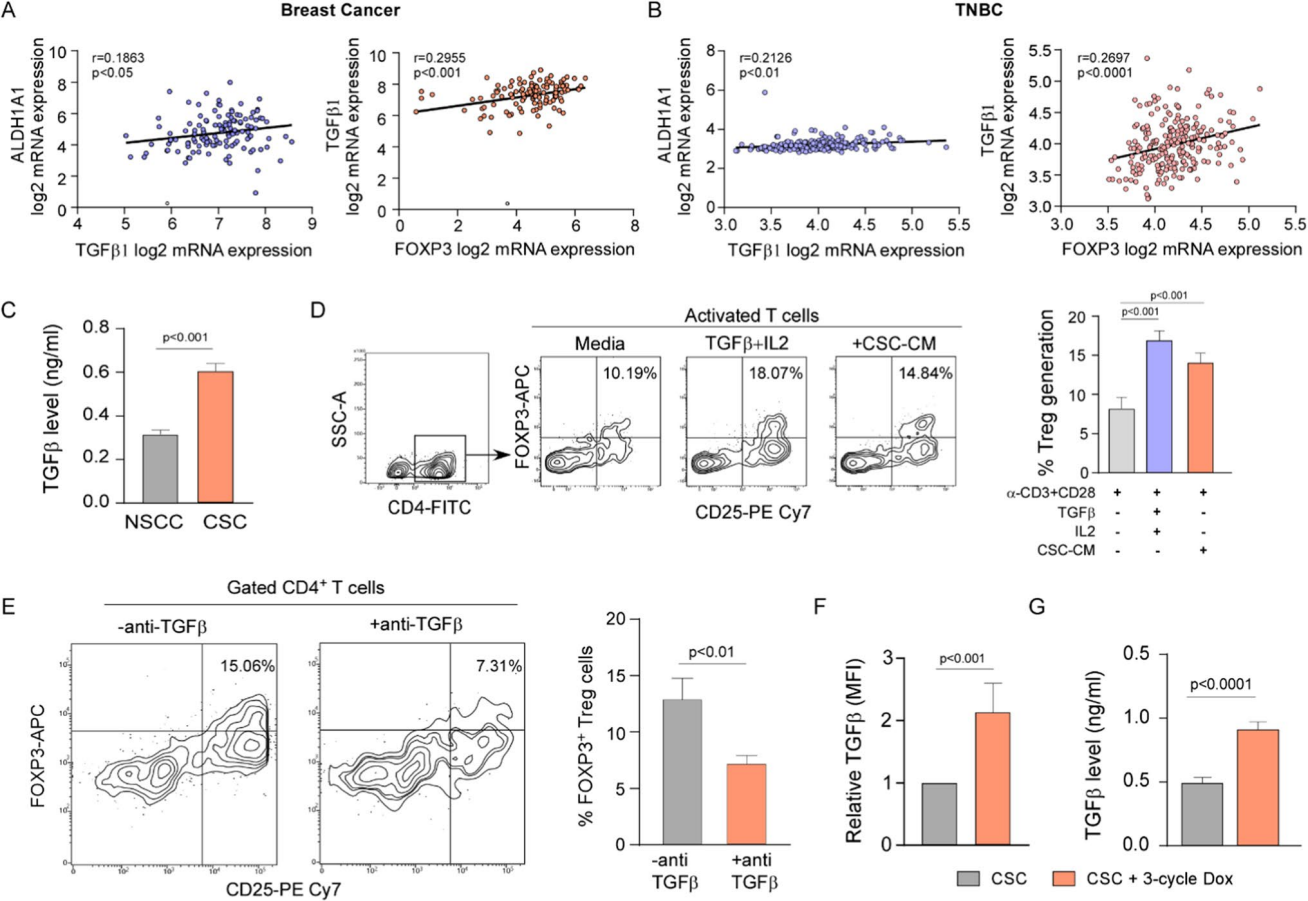
CSC-secreted TGFβ induces immunosuppressive Treg cell generation. **A** Plot showing positive correlation between CSC markers ALDH1A1 and TGFβ1, from GSE69031 breast cancer patient dataset (left) and representative plot demonstrating correlation between TGFβ1 and suppressive Treg marker FOXP3 using GSE5460 breast cancer dataset (right panel). Datasets were obtained from ‘R2: Genomics Analysis and Visualization Platform’ database. “r” is correlation co-efficient. **B** Plots showing a positive correlation of ALDH1A1 with TGFβ (left panel) and FOXP3 with TGFβ (right panel) in TNBC dataset (GSE142102) available in ‘R2: Genomics Analysis and Visualization Platform’. **C** Representative bar plots showing level of secreted TGFβ in the CM of MDA-MB-468-derived CSCs and NSCCs, as determined by ELISA. **D** FACS plots (left panel) and bar diagram (right panel) depicting percent immune-suppressive Treg cells generated from CD4^+^ T cells following activation by (i) only α-CD3 + α-CD28, (ii) α-CD3 + α-CD28 + TGFβ + IL2, and (iii) α-CD3 + α-CD28 + CSC-CM. **E** FACS plots (left panel) and bar diagram (right panel) depicting percent Treg cell generation from activated T cells cultured in presence of CSC-CM pre-incubated with or without anti-TGFβ antibody. **F** Bar plot demonstrating expression of TGFβ per-CSC in control vs. 3-cycles of doxorubicin-treated CSCs derived from MDA-MB-468 spheroids. **G** Representative bar plot showing the amount of TGFβ present in the CM of control vs. 3-cycles of doxorubicin-treated CSCs derived from MDA-MB-468 spheroids determined by ELISA. Data were represented as the mean ± SD of minimum 3 independent experiments performed in triplicate. Student’s t-test (unpaired) was used to assess the data where *P < 0.05, **P < 0.01, ***P < 0.001, and ****P < 0.0001. CSC: cancer stem cell; MFI: mean fluorescence intensity; CM: conditioned medium

To that end, supporting our hypothesis, in-silico RNA profiling database from ‘R2: Genomics Analysis and Visualization Platform’ showed a positive correlation between CSC marker ALDH1A1 and TGFβ1 (GSE 69,031) (r = 0.1863; p < 0.05) (Fig. 4A left panel) as well as Treg signature FOXP3 and TGFβ1 (GSE5460) (r = 0.2955; p < 0.001) (Fig. 4A right panel) in breast cancer patients. Again, ‘R2: Genomics Analysis and Visualization Platform’ database analysis revealed a positive correlation of ALDH1A1 with TGFβ (r = 0.2126, p < 0.01, n = 226) and FOXP3 with TGFβ (r = 0.2697, p < 0.0001, n = 226) in TNBC subset (GSE142102) as well (Fig. 4B).

Furthermore, according to Shipitsin et al. TGFβ is up-regulated in CD44^+^-expressing breast cancer cells [61]. Our ELISA results depicted that TGFβ level was significantly higher in MDA-MB-468 CSC-CM than NSCC-CM (p < 0.001) (Fig. 4C). Next, anti-CD3/anti-CD28-treated T cells were exposed to (i) CM of Miltenyi bead purified CSC (CSC: T cell ratio 1:5) or (ii) stimulation by IL2 and TGFβ [27]. We observed that, CSC-CM could generate ~ 15% Treg cells (which was comparable to that formed (~ 18%) upon stimulation by IL2 and TGFβ, taken as positive control) in comparison to anti-CD3/anti-CD28-treated CD4^+^ T cells (Fig. 4D).

Since we have confirmed earlier that our CSC-CM is enriched in TGFβ level (Fig. 4C), this observation directed us to hypothesize that CSC-shed TGFβ might be involved in the augmented generation of Treg cells. However, possibility of the contribution of IL2, if at all secreted by CSCs, has not been negated. Finally, to re-affirm the contribution of CSC-shed TGFβ in Treg formation, when we neutralized TGFβ in CSC-CM, a significant decrease (p < 0.01) in Treg percentage, validating the essential role of CSC-released TGFβ in Treg cell generation (CSC: T cell ratio 1:5) (Fig. 4E).

### Chemotherapy further increases TGFβ in CSCs thus resulting in a higher Treg polarization favoring tumor relapse

Above results together tempted us to hypothesize that chemotherapy might further increase TGFβ in CSCs thereby resulting in a higher Treg polarization and culminating in a microenvironment that favors tumor relapse. To validate our hypothesis, TGFβ expression was evaluated flow-cytometrically in the isolated CD44^+^/CD24^−^ CSC population in presence of PMA + Ionomycin + Brefeldin A (last 5 h of culture) with or without multiple rounds of doxorubicin treatment. A significant (p < 0.001) increase in TGFβ content (~ 2.2 fold) after the third cycle of doxorubicin-treated MDA-MB-468 CSCs was obtained in comparison to untreated CSCs (Fig. 4F). To revalidate these results, we next checked secretory TGFβ levels in the CM of above-mentioned cells by ELISA and found that 3 cycles of chemotherapy significantly (p < 0.0001) increased CSC-shed TGFβ in the CM than the same collected from non-treated CSCs (Fig. 4G).

These results signify that chemotherapy-escaped CSCs, by secreting more TGFβ, generate more Treg cells than the untreated tumor-initiating CSCs, thereby facilitating immune-evasion and ensuring recurrence even after a few months to several years [9].

This shows how CSCs even present in low numbers during tumor initiating or relapse phase, are able to evade immune destruction by converting CD4^+^ T cells to immunosuppressive Treg cells. It would not be out of context to mention at this point that at tumor initiating conditions, expression of IFNγ, a key Th1 response cytokine [62] was suppressed when anti-CD3/anti-CD28-treated CD4^+^ T cells were exposed to CM of MDA-MB-468 CSC (CSC: T cell ratio 1:5) in comparison to unexposed anti-CD3/anti-CD28-treated CD4^+^ T cells (p < 0.01) (Online Resource 5, Supplementary Fig. 3). However, as compared to control CSC-CM, when TGFβ was neutralized in CSC-CM, an increase in IFNγ expression was noted in the activated T cell subset (p < 0.05), validating the role of CSC-shed TGFβ in decreasing Th1 response. Additionally, recombinant TGFβ, when directly added to activated CD4^+^ T cells, decreased IFNγ levels (p < 0.001) (Online Resource 5, Supplementary Fig. 3). These findings signify the contribution of CSC-shed TGFβ in creating an immunosuppressive environment in more ways than one, to ensure proper tumor development.

## Discussion

Interaction between the myriad of cells present in the TME effectively decides the fate of tumor progression. On the other hand, CSCs play pivotal role in forming a heterogenous tumor mass during the course of tumor initiation [3]. This tumor-initiating property of CSCs signifies their candidature as the initiators of new tumors during metastatic relapse [63] at distant organs, and recurrence after removal of chemotherapy due to survival of such drug-spared CSCs [8, 49]. However, during all such initiation phases, it becomes crucial for CSCs to evade immune-elimination phase by modulating the TME thus initiating tumorigenesis. A better knowledge about the mechanisms underneath immuno-editing by CSCs that help them escaping initial ‘elimination’ by surrounding immune cells, is thus essential for designing more effective therapies.

Although innumerable reports have comprehensively provided information about the interactions of tumor cells with immune system within the TME, the investigation of CSC-immune dynamics has only initiated to reveal, paving the path for exploring the potential therapeutic regimen. However, the ability of CSCs over NSCCs to initiate tumor in partially immuno-compromised mice [64] indicate that CSCs might be endowed with higher ability to escape immune recognition and surveillance. However, there is dearth of detail information regarding CSC-driven immune-modulation, fine and timely interception of which might open new avenues to precision medicine. Recruitment of immune-suppressive Tregs and interestingly, polarizing effector CD4^+ ^T cells to pro-tumor Treg subset demonstrates the immune-modulatory feature of the TME [65, 66]. Albeit present in low number [40, 41] even without the NSCCs, how CSCs modulate effector immune cells during the phases of early initiation and recurrence, remain elusive.

Our exploration revealing the positive correlation between occurrence of CSCs and Tregs encouraged us to further explore whether CSCs even though present in low numbers, can generate CD4^+^CD25^+^FOXP3^+^ immunosuppressive Treg cells from effector CD4^+^CD25^−^ T lymphocytes during tumor initiation phase. Our results being in line with our hypothesis, foster the strong immune-regulatory effect of CSCs at the beginning of tumor formation, resulting in self-endurance along with genesis of NSCCs and the entire tumor mass. Tregs in turn encourage tumor progression by releasing cytokines like TGFβ [67, 68] as well as IL17 [69–71], aiding in EMT and maintaining stemness [67, 68, 72], and under hypoxic conditions supporting angiogenesis by secreting VEGF [73]. Furthermore, Treg accumulation in breast cancer patients down-regulates effector T cell proliferation and pro-inflammatory factors such as IFNγ [46], thus systematically altering the immune response toward an anti-inflammatory, pro-tumorigenic phenotype facilitating tumor metastasis. CXCR4^+^ metastatic CSCs, known to seed tumors at distant organs [6] may follow this probable mode of immunomodulatory action as seen in initiating conditions.

On the other hand, during tumor relapse, CSCs escape chemotherapy and increase in number [8, 47] due to their ability to transit into quiescent cell phase [74] as well as their high expression of multi-drug resistance proteins [48]. However, this may not fully explain why even after NACT, tumor relapses at a distant organ. Interestingly, previous studies point out that CSCs migrate to distant sites post-chemotherapy for their sustenance [8]. Therefore, this explains why breast cancer patients undergoing NACT showed a correlation between high stemness factors and low relapse-free survival. Cumulatively, CSCs are responsible for therapy resistance and tumor recurrence, wherein they ingrain an entire tumor mass either in the same organ or at distant organs, although present in tiny amount. Concurrently, it was also reflected in our findings that during multiple rounds of chemotherapeutic stress, majority of the NSCC population succumbed, however, the drug failed to exert any significant cytolytic/apoptotic effect on CSCs, rather the CSC repertoire was increased. Moreover, isolated CSCs from the persevering population managed to generate higher Treg cells as well, when compared to Treg generation potential from the same number of untreated CSCs in our study. Number of CSCs being same, these results point towards the possibility of chemotherapy-induced enhancement of CSC properties. Augmented Treg numbers during chemotherapy also clarifies why higher FOXP3 expression correlates with lower RFS in NACT-treated breast cancer patients as observed in our in-silico data. Further validating our findings, high Treg activity has been reported to be a predicting factor for future tumor recurrence [75] as well as distant metastatic relapse [76]. These results along with the literature search together illuminate how after chemotherapy, aggressive CSCs again evade the immune-elimination with the help of CSC-induced Tregs, so that they can later emerge and recreate the tumor.

At this juncture, our literature survey showed the vital implication of TGFβ in Treg generation [56]. Both cancer cells [50] as well as CSCs [61] have been reported to secrete TGFβ. Additionally, that cancer cell-shed TGFβ generate Tregs from naïve T cells [24, 60] reinforced our line of thought. These reports and our in-silico evidences depicting a positive relationship between CSCs and Treg with TGFβ, tempted us to investigate the conditions of CSC-induced Treg generation during primary initiation and during relapse after chemotherapy, which have been studied limitedly. CSCs were found to secrete higher amount of TGFβ compared to NSCCs, justifying the higher Treg generation in case of CSCs to ensure their own survival for tumor initiation when even NSCCs are not present. At this point, it may be mentioned that not only TGFβ, CSCs also shed higher levels of IL6 than NSCCs [8]. Further reports depicted the contribution of IL6 in generating RORγt ^+^ Tregs [77, 78]. This information, together, point towards another possible mechanism of immunosuppression by CSCs.

Interestingly, chemotherapy-treated CSCs secrete even higher amount of TGFβ than same number of untreated CSCs. Reports are there signifying up-regulated levels of TGFβ gene score and expression upon chemotherapy [50]. These results translate the idea that doxorubicin enhances the CSC properties resulting in augmented release of TGFβ by them. In fact, our previous lab finding reported heightened IL6 expression in chemo-treated CSCs than untreated CSCs [8], confirming chemo-treatment can potentiate CSCs to release higher pro-tumor cytokines. Thereby, our results furnishing increased stemness per-CSC after chemotherapy further strengthen our hypothesis. These findings and the previous reports together confirm that CSCs evade immune-attack by converting anti-tumor T cells to pro-tumor Treg cells via generation of immune-suppressive cytokine TGFβ, to initiate tumor either at the primary site or during tumor recurrence following withdrawal of chemotherapy.

In addition to inducing an immunosuppressive phenotype, CSC-shed TGFβ is also reported to suppress Th1 response as well, thereby, eliciting a pro-tumor environment in a concerted manner. Head and neck carcinoma CD44^+^ CSCs containing elevated levels of various cytokines, including TGFβ, has been reported to inhibit production of IFNγ [79], a key marker of Th1 response [62]. Furthermore, TGFβ itself suppressed the expression of IFNγ and TNFα in PBMCs [80], both of which are considered key cytokines of Th1 response [62]. Concurrently, we too observed that CSC-shed TGFβ was able to curb IFNγ expression in the activated CD4^+^ T cell subset. This discussion further highlights the importance of CSC-shed TGFβ in creating an immunosuppressive environment by multiple ways during tumor initiation and relapse conditions.

In conclusion, CSCs effectively and actively manipulate immune system in their favor to protect these tumor-initiators from immune-insult, ensuring tumor initiation. In fact, to avoid the threat of direct physical interaction with anti-tumor T cells, CSCs, although being present in low numbers both during tumor initiation and relapse, judiciously generate Treg cells in contact-independent manner by shedding TGFβ that in turn results in effective Treg polarization, to curb effector T cells. Our study demonstrates for the first time, how CSCs evade the immune surveillance to initiate tumor during primary tumor initiation as well as during tumor relapse after chemotherapy, thus paving way for future research in developing successful CSC-targeted immunotherapy to eradicate cancer.

### Electronic supplementary material

Below is the link to the electronic supplementary material.

Supplementary Material 1

Supplementary Material 2

Supplementary Material 3

Supplementary Material 4

Supplementary Material 5

## Data Availability

The datasets generated during and/or analyzed during the current study are available from the corresponding author on reasonable request.
