# Supplementary material for: Breast cancer stem cells generate immune-suppressive T regulatory cells by secreting TGFβ to evade immune-elimination
Source: Discov Oncol. 2023 Dec 1;14:220. doi: 10.1007/s12672-023-00787-z (PMC10692020; doi:10.1007/s12672-023-00787-z)
Supplement: Supplementary file 1 — Supplementary Material 1 [file 12672_2023_787_MOESM1_ESM.docx]

**Discover Oncology**

**Breast cancer stem cells generate immune-suppressive T regulatory cells by secreting TGFβ to evade immune-elimination**

**Sumon Mukherjee^1t^, Sourio Chakraborty^1t^, Udit Basak^1t^, Subhadip Pati^1^, Apratim Dutta^1^, Saikat Dutta^1^, Dia Roy^1^, Shruti Banerjee^1^, Arpan Ray^2^, Gaurisankar Sa^1^, and Tanya Das^1*^**

^1^Division of Molecular Medicine, Bose Institute, P-1/12, Calcutta Improvement Trust Scheme VII M, Kolkata-700054, India.

^2^Department of Pathology, ESI-PGIMSR, Medical College Hospital and ODC (EZ), Kolkata, India.

^t^SM, SC and UB have contributed equally

*For correspondence: [tanya@jcbose.ac.in](mailto:tanya@jcbose.ac.in), das_tanya@yahoo.com

**Supplementary Table 1. Details of the breast cancer (BC) patients enrolled in the study**

| **No.** | **Age(years)** | **Sex** | **NACT** | **ER** | **PR** | **Her2** | **Pathologic**  **stage** | **Histologic**  **grade** |
| --- | --- | --- | --- | --- | --- | --- | --- | --- |
| 1. | 60 | F | No | Negative | Negative | Negative | NA | NA |
| 2. | 44 | F | No | Negative | Negative | Positive | T4N1 | 2 |
| 3. | 48 | F | No | Negative | Negative | Negative | T3N0 | 3 |
| 4. | 37 | F | Yes | Negative | Negative | Negative | T1N0 | 2 |
| 5. | 48 | F | Yes | Positive | Positive | Negative | T4N3 | 3 |
| 6. | 36 | F | No | Negative | Negative | Negative | T2N2 | 3 |
| 7. | 45 | F | Yes | Negative | Negative | Negative | T2N0 | 3 |
| 8. | 37 | F | No | Positive | Positive | Negative | T2N3 | 2 |
| 9. | 54 | F | No | Negative | Negative | Positive | T2N1 | 3 |
| 10. | 59 | F | No | Negative | Negative | Negative | T2N0 | 3 |
| 11. | 65 | F | No | Negative | Negative | Negative | T2N0 | 3 |
| 12. | 47 | F | Yes | Negative | Negative | Negative | T3N2 | 3 |
| 13. | 58 | F | No | Positive | Positive | Negative | T4N3 | 2 |
| 14. | 48 | F | Yes | Negative | Negative | Positive | T3N1 | 3 |
| 15. | 60 | F | No | Positive | Positive | Negative | T2N0 | 2 |
| 16. | 39 | F | No | Positive | Positive | Negative | T3N1 | 2 |
| 17. | 60 | F | No | Positive | Positive | Negative | NA | 3 |
| 18. | 49 | F | No | Positive | Positive | Negative | T2N3 | 2 |
