# Supplementary material for: Breast cancer stem cells generate immune-suppressive T regulatory cells by secreting TGFβ to evade immune-elimination
Source: Discov Oncol. 2023 Dec 1;14:220. doi: 10.1007/s12672-023-00787-z (PMC10692020; doi:10.1007/s12672-023-00787-z)
Supplement: Supplementary file 2 — Supplementary Material 2 [file 12672_2023_787_MOESM2_ESM.docx]

**Discover Oncology**

**Breast cancer stem cells generate immune-suppressive T regulatory cells by secreting TGFβ to evade immune-elimination**

**Sumon Mukherjee^1t^, Sourio Chakraborty^1t^, Udit Basak^1t^, Subhadip Pati^1^, Apratim Dutta^1^, Saikat Dutta^1^, Dia Roy^1^, Shruti Banerjee^1^, Arpan Ray^2^, Gaurisankar Sa^1^, and Tanya Das^1*^**

^1^Division of Molecular Medicine, Bose Institute, P-1/12, Calcutta Improvement Trust Scheme VII M, Kolkata-700054, India.

^2^Department of Pathology, ESI-PGIMSR, Medical College Hospital and ODC (EZ), Kolkata, India.

^t^SM, SC and UB have contributed equally

*For correspondence: [tanya@jcbose.ac.in](mailto:tanya@jcbose.ac.in), das_tanya@yahoo.com

**Supplementary Table 2. Details of the fluorophore-conjugated antibodies used in flow-cytometry**

| **Antibodies** | **Source** | **Cat. No.** |
| --- | --- | --- |
| CD4-APC H7 | BD Biosceiences | 560158 |
| CD4-FITC | Biolegend | 317408 |
| CD25-PE Cy7 | Biolegend | 302612 |
| FOXP3-APC | BD pharmingen | 560889 |
| CD127-APC | BD Pharmingen | 558598 |
| TGFβ-BV421 | BD Horizon | 562962 |
| CD44-APC | BD Pharmingen | 559942 |
| CD24-PE | BD Pharmingen | 555428 |
| Annexin V-FITC | BD Pharmingen | 560931 |
| OCT4-PerCP-Cy5.5 | BD Stemflow | 51-9006267 |
| SOX2-APC | BD Stemflow | 51-9006407 |
| NANOG-PE | BD Stemflow | 51-9006414 |
| IFNγ-PE | BD Pharmingen | 562016 |
| CFSE | Biolegend | 423801 |
| CD127-PerCP Cy5.5 | BD Pharmingen | 560551 |
