# Supplementary material for: Breast cancer stem cells generate immune-suppressive T regulatory cells by secreting TGFβ to evade immune-elimination
Source: Discov Oncol. 2023 Dec 1;14:220. doi: 10.1007/s12672-023-00787-z (PMC10692020; doi:10.1007/s12672-023-00787-z)
Supplement: Supplementary file 3 — Supplementary Material 3 [file 12672_2023_787_MOESM3_ESM.docx]

**Discover Oncology**

**Breast cancer stem cells generate immune-suppressive T regulatory cells by secreting TGFβ to evade immune-elimination**

**Sumon Mukherjee^1t^, Sourio Chakraborty^1t^, Udit Basak^1t^, Subhadip Pati^1^, Apratim Dutta^1^, Saikat Dutta^1^, Dia Roy^1^, Shruti Banerjee^1^, Arpan Ray^2^, Gaurisankar Sa^1^, and Tanya Das^1*^**

^1^Division of Molecular Medicine, Bose Institute, P-1/12, Calcutta Improvement Trust Scheme VII M, Kolkata-700054, India.

^2^Department of Pathology, ESI-PGIMSR, Medical College Hospital and ODC (EZ), Kolkata, India.

^t^SM, SC and UB have contributed equally

*For correspondence: [tanya@jcbose.ac.in](mailto:tanya@jcbose.ac.in), das_tanya@yahoo.com

**Supplementary Figure 1.**


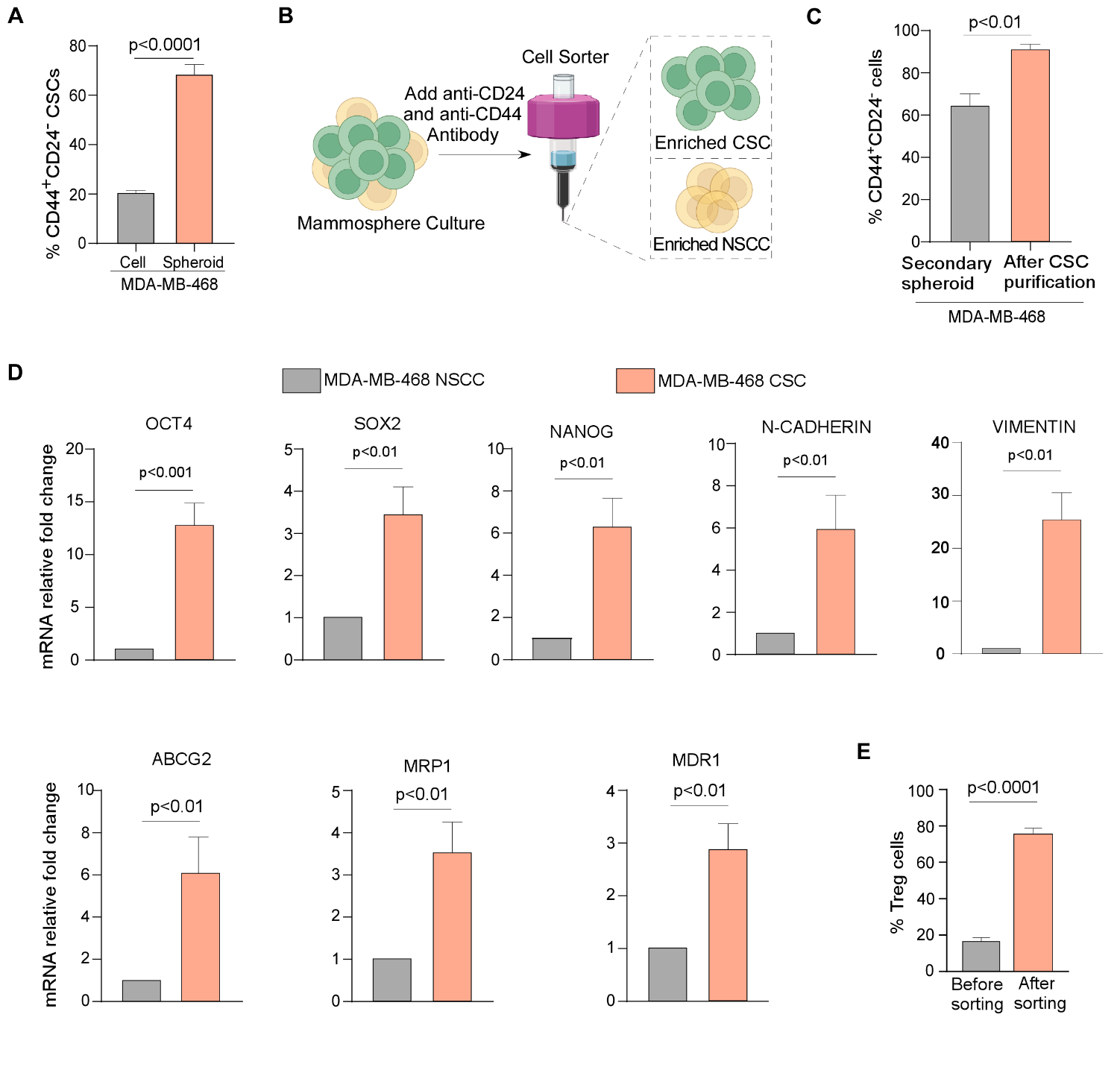


### Supplementary Fig. 1: (A) Bar graph showing percent CD44^+^/CD24^-^ cell population in monolayer MDA-MB-468 cell vs. MDA-MB-468 cell-derived spheroids, as determined by flow-cytometry. (B) Schematic representation of Miltenyi MACS bead-based sorting of CD44^+^/CD24^-^ CSC and CD44^-^/CD24^-^, CD44^-^/CD24^+^, CD44^+^/CD24^+^ NSCC population from MDA-MB-468 cell-derived spheroids. (C) Bar diagram depicting percent of CD44^+^/CD24^-^ CSC population in MDA-MB-468 cell-derived spheroids before and after their MACS bead-based separation as determined by flow-cytometry. (D) Bar graphs demonstrating the relative mRNA expression of stemness markers OCT4, SOX2, and NANOG; EMT markers N-CADHERIN, and VIMENTIN; and drug-resistance markers ABCG2, MRP1, and MDR1 in isolated NSCC vs. CSC population from MDA-MB-468 cell-derived spheroids, as determined by qRT-PCR assay. 18S rRNA gene was used as the constitutively expressed gene. (E) Bar diagram showing percentage of CSC-CM-derived Treg cells before and after their magnetic column-based sorting, as determined by flow-cytometry. Data were represented as the mean ± SD of minimum 3 independent experiments performed in triplicate. Student’s t-test (unpaired) was used to assess the data where *P < 0.05, **P < 0.01, ***P < 0.001, and ****P < 0.0001.CSC: cancer stem cell; NSCC: non-stem cancer cell.
