# Supplementary material for: Breast cancer stem cells generate immune-suppressive T regulatory cells by secreting TGFβ to evade immune-elimination
Source: Discov Oncol. 2023 Dec 1;14:220. doi: 10.1007/s12672-023-00787-z (PMC10692020; doi:10.1007/s12672-023-00787-z)
Supplement: Supplementary file 4 — Supplementary Material 4 [file 12672_2023_787_MOESM4_ESM.docx]

**Discover Oncology**

**Breast cancer stem cells generate immune-suppressive T regulatory cells by secreting TGFβ to evade immune-elimination**

**Sumon Mukherjee^1t^, Sourio Chakraborty^1t^, Udit Basak^1t^, Subhadip Pati^1^, Apratim Dutta^1^, Saikat Dutta^1^, Dia Roy^1^, Shruti Banerjee^1^, Arpan Ray^2^, Gaurisankar Sa^1^, and Tanya Das^1*^**

^1^Division of Molecular Medicine, Bose Institute, P-1/12, Calcutta Improvement Trust Scheme VII M, Kolkata-700054, India.

^2^Department of Pathology, ESI-PGIMSR, Medical College Hospital and ODC (EZ), Kolkata, India.

^t^SM, SC and UB have contributed equally

*For correspondence: [tanya@jcbose.ac.in](mailto:tanya@jcbose.ac.in), das_tanya@yahoo.com

**Supplementary Figure 2.**


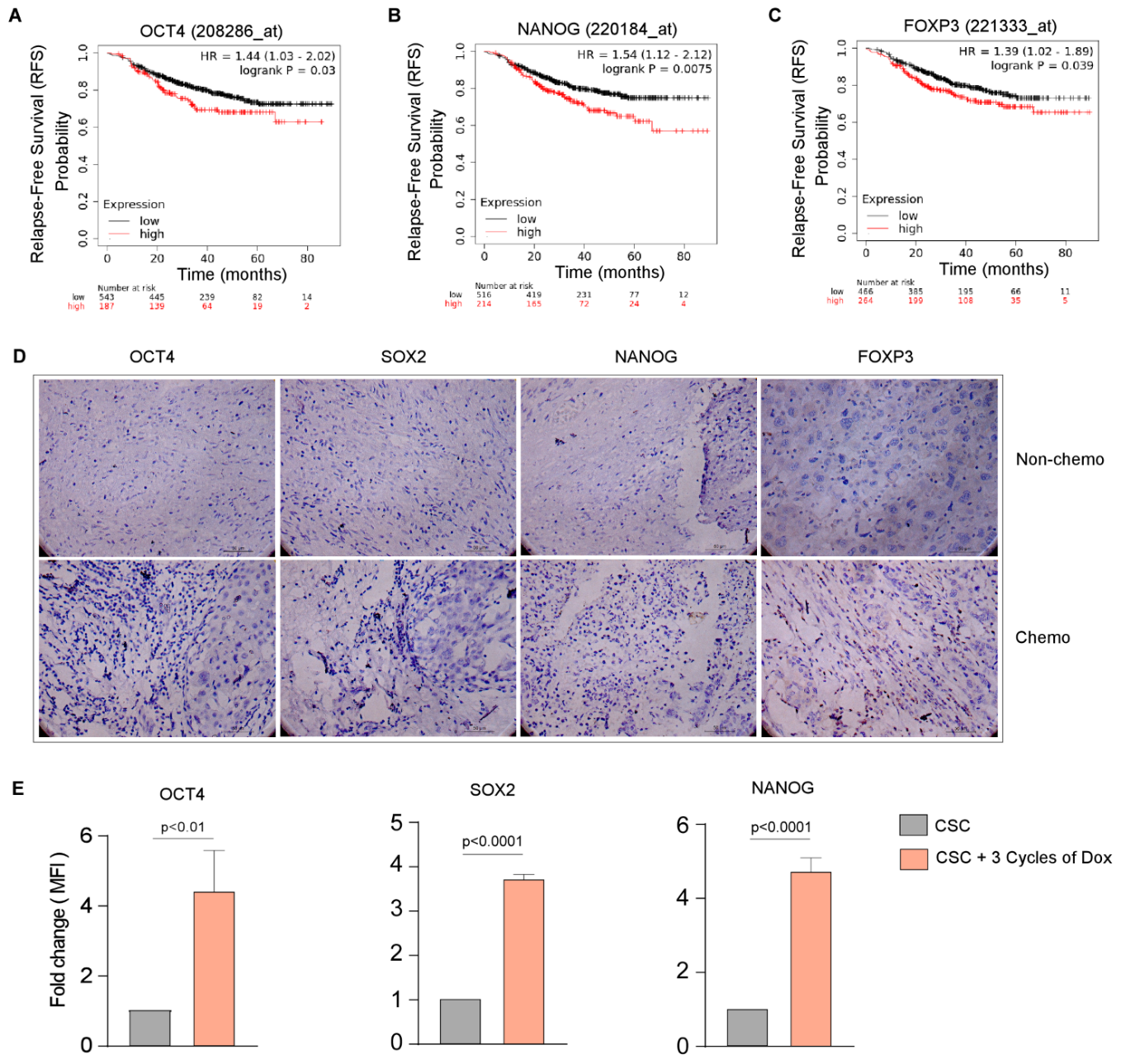


**Supplementary Fig. 2:** Kaplan-Meier (KM) plots showing reduced relapse-free survival (RFS) of breast tumor patients harboring high (A) OCT4, (B) NANOG, and (C) FOXP3 expression, after undergoing NACT. (D) Representative immunohistochemistry (IHC) images showing augmented number of OCT4 (left panel), SOX2 (middle panel), NANOG (right panel), and FOXP3 (right most panel) containing cells in chemotherapy-treated breast cancer patient-derived tissues than non-treated breast cancer patient-derived tissues (n=5 from each group). Scale bar = 50 μM and magnification 40X. (E) Bar graphs showing levels of stemness factors OCT4, SOX2, and NANOG in control vs. 3-cycles of doxorubicin-treated MDA-MB-468 spheroid-derived CSCs as determined by flow-cytometry. Data were represented as the mean ± SD of minimum 3 independent experiments performed in triplicate. Student’s t-test (unpaired) was used to assess the data where *P < 0.05, **P < 0.01, ***P < 0.001, and ****P < 0.0001. CSC: cancer stem cell; non-chemo: chemotherapy untreated; chemo: chemotherapy-treated; NACT: neo-adjuvant chemotherapy.
