# Supplementary material for: Breast cancer stem cells generate immune-suppressive T regulatory cells by secreting TGFβ to evade immune-elimination
Source: Discov Oncol. 2023 Dec 1;14:220. doi: 10.1007/s12672-023-00787-z (PMC10692020; doi:10.1007/s12672-023-00787-z)
Supplement: Supplementary file 5 — Supplementary Material 5 [file 12672_2023_787_MOESM5_ESM.docx]

**Discover Oncology**

**Breast cancer stem cells generate immune-suppressive T regulatory cells by secreting TGFβ to evade immune-elimination**

**Sumon Mukherjee^1t^, Sourio Chakraborty^1t^, Udit Basak^1t^, Subhadip Pati^1^, Apratim Dutta^1^, Saikat Dutta^1^, Dia Roy^1^, Shruti Banerjee^1^, Arpan Ray^2^, Gaurisankar Sa^1^, and Tanya Das^1*^**

^1^Division of Molecular Medicine, Bose Institute, P-1/12, Calcutta Improvement Trust Scheme VII M, Kolkata-700054, India.

^2^Department of Pathology, ESI-PGIMSR, Medical College Hospital and ODC (EZ), Kolkata, India.

^t^SM, SC and UB have contributed equally

*For correspondence: [tanya@jcbose.ac.in](mailto:tanya@jcbose.ac.in), das_tanya@yahoo.com

**Supplementary Figure 3.**

**
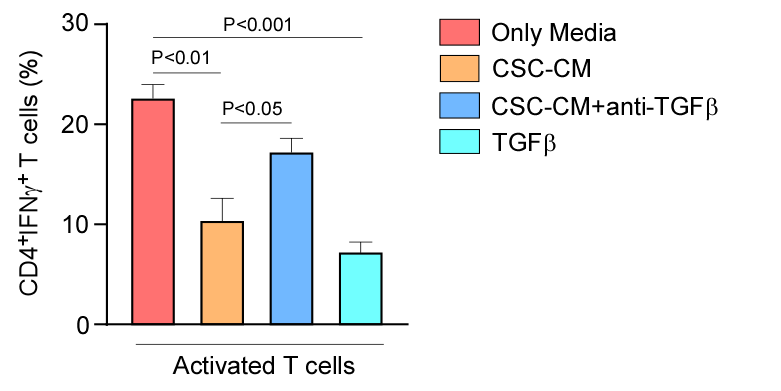
**

**Supplementary Fig. 3**: Representative bar graph showing percentage of IFNγ expression in CD4^+^ T cells under the following conditions: (i) only α-CD3/α-CD28, (ii) α-CD3/α-CD28 + CSC-CM, and (iii) α-CD3/α-CD28 + CSC-CM + TGFβ neutralizing Ab (1μg/ml) (iv) α-CD3/α-CD28 + recombinant TGFβ (5ng/ml). Data were represented as the mean ± SD of minimum 3 independent experiments performed in triplicate. Student’s t-test (unpaired) was used to assess the data where *P < 0.05, **P < 0.01, ***P < 0.001, and ****P < 0.0001.
